# Knowledge Level on Infection Control among Romanian Undergraduate and Postgraduate Dental Students

**DOI:** 10.3390/medicina58050661

**Published:** 2022-05-13

**Authors:** Catalina Iulia Saveanu, Maria Diana Meslec, Alexandra Ecaterina Saveanu, Daniela Anistoroaei, Livia Bobu, Carina Balcos, Oana Tanculescu

**Affiliations:** 1 Surgicals Department, Faculty of Dental Medicine, Grigore T. Popa University of Medicine and Pharmacy, 700115 Iasi, Romania; cisaveanu@prevod.umfiasi.ro (C.I.S.); carina.balcos@umfiasi.ro (C.B.); 2 Department of Dent-Alveolar and Maxillo-Facial Surgery, Faculty of Dental Medicine, Grigore T. Popa University of Medicine and Pharmacy, 700115 Iasi, Romania; diana.meslec@yahoo.com (M.D.M.); alexandra-ecaterina.dd.saveanu@students.umfiasi.ro (A.E.S.); 3 Department of Fixed Prosthodontics, Faculty of Dental Medicine, Grigore T. Popa University of Medicine and Pharmacy, 700115 Iasi, Romania; ot@umfiasi.ro

**Keywords:** infection control, hand hygiene, hand pieces, high-level disinfection, sterilization

## Abstract

*Background and Objectives*: Infection control practices in dentistry should be continuously evaluated. The aim of the present study was to assess the knowledge of dental students from Iași, Romania about infection control in the dental office. *Materials and Methods*: Dental students and resident dentists attending the “Grigore T. Popa” University of Medicine and Pharmacy in Iași were randomly selected in accordance with ethical guidelines, and a cross-sectional, questionnaire-based online study was conducted. The questionnaire included 21 items about infection control in dentistry. A descriptive statistical analysis was performed, and the chi-square test was used for data comparison, with a cutoff point of 0.05 for statistical significance. *Results*: The study sample included 150 subjects (75.3% female and 24.7% male) with a mean age of 25.71 ± 4.54 years. Mode of infection transmission was known by 74% of the subjects, and 76% were aware of standard precautions, with significant differences by the year of study (*p* = 0.012, *r* = 0.002). A percentage of 20% of subjects knew the means of transmission of the hepatitis B virus (HBV) (*p* = 0.032, *r* = 0.166). Most of the subjects were not vaccinated against HBV (*p* = 0.002, *r* = −0.274). Notions of high-level disinfection and sterilization were confused by 19.5% of the subjects. Only 22% of the subjects knew the correct processing of handpieces (*p* = 0.048, *r* = −0.071). The sources of information were diverse for 64.66% of the respondents, while 31.33% of them used courses and seminars only. *Conclusions*: There is a need for improvement in the level of knowledge on infection control for both dental students and residents.

## 1. Introduction

Infection control should be one of the main desiderata of the dental clinical practice. Measures have to be taken to reduce the risk of exposure associated with dental health services, and infection control practices should be continuously evaluated. It is also very important for all dentists to be up to date with the specifications of the Centers for Disease Control and Prevention, and with the equipment and techniques recommended for adequate infection control for both patients and medical staff [1]. Basically, in terms of infection control, dentists and other healthcare workers have ethical, professional, and legal obligations to fulfill. Patients want a safe environment for their dental healthcare. Dental students should learn the importance of infection control during their university training so that they adopt the right attitudes and behaviors as dental professionals. Faculties of dentistry are responsible for implementing appropriate infection control strategies and supporting student immunization programs. Education plays an important role in providing appropriate knowledge and attitudes related to infection control measures. Methods of assessing knowledge, such as programs or courses on infection control, should be implemented so that dental students, as well as dentists, are updated with new techniques and are aware of the importance of measures to prevent infection transmission, thus offering the safest and highest quality medical services [2,3,4,5].

Cross-infection and infection control in dentistry are topical issues. Knowing the chain of infection and breaking one of its links are essential requirements for disease prevention, especially in a pandemic context [6,7]. A review on this topic showed that high standards of infection control measures are of utmost importance for dental healthcare workers to avoid infectious disease transmission via cross-contamination [8].

Hand hygiene (HH) is the basis of infection control programs [9,10,11,12,13,14,15,16]. Numerous studies have shown gaps in hand hygiene behaviors, which are also highlighted in the context of the SARS-CoV-2 pandemic [17,18,19,20]. The use of protective gloves does not provide absolute protection. Therefore, HH is very important. HH with antiseptic solution is very frequently used in daily practice and, although it is effective on transient microbial flora and resident microbial flora, it is not effective in removing organic matter [21]. For this reason, some researchers consider HH with soap and water to be more effective than decontamination with antiseptic solutions [22]. Although the recommendations of the World Health Organization (WHO) Hand Hygiene Guide contain all the necessary guidelines, practitioners are not sufficiently informed or do not adopt these routine procedures correctly [11].

Prevention of airborne infection transmission is a topical concern for specialists in the field, and all dental professionals should be aware of the aerosol-generating dental procedures. A recent study has shown that the disinfectants that best suit the needs of the dental clinic are hydrogen peroxide and sodium hypochlorite [23]. Preprocedural mouthrinses and the use of a rubber dam can lead to a considerable reduction of airborne contaminants. Therefore, knowledge of the protocol and its application are necessary. Dental offices and dental staff can become a dangerous source of airborne disease transmission unless proper infection control measures are implemented [24]. Numerous studies have been conducted on the effectiveness of protective masks [25,26,27,28,29]. The protective mask provides protection against airborne contaminants, but it retains its maximum effectiveness only for the first 30 min after application (in the case of surgical masks), which should be known by all practitioners [30].

The Occupational Safety and Health Administration (OSHA)’s bloodborne standard for reducing exposure risk and for infection control is represented by the adoption of Universal Precautions. Employees who observe Universal Precautions will treat all potentially infectious materials with appropriate precautions, such as hand hygiene, the use of personal protective equipment (PPE), and tools and work practice controls to limit exposure [31]. Bloodborne pathogens raise a serious risk to healthcare workers. Of the 20 bloodborne pathogens, hepatitis B virus (HBV), hepatitis C virus (HCV) and human immunodeficiency virus (HIV) account for the majority of professionally acquired infections and are associated with significant morbidity and mortality. The HBV vaccine has been available since 1981 and is mandatory for all employees at risk of occupational exposure. However, HBV has a risk of transmission of 30% in the dental office. Due to the high prevalence and the constantly increasing incidence among health workers, rules and standards are essential to control the infection. The risk of HCV transmission when dental staff are exposed to HCV-positive blood is 1.8%, which is considerably lower than HBV, but there is currently no vaccine or postexposure prophylaxis (PEP) for HCV infection [31]. The percutaneous risk of HIV transmission in the dental office is estimated at about 0.3%, the lowest when compared to HBV and HCV. Proper application of postexposure protocols to infected blood is especially important. For example, in the case of exposure to HIV-infected blood, postexposure prophylactic drugs are very effective in infection prevention, provided the treatment is administered within the first 72 h [31].

The control plan for the prevention of exposure to a pathogen primarily involves the training of personnel. Staff training should primarily include ways to reduce the risk of transmission of blood-borne pathogens. In conjunction with these notions, each worker in the field should be familiar with the series of hepatitis B vaccines; the recognition of high-risk exposure situation; methods of reducing exposure; working control practices; and proper use and selection of PPE, including removal, handling, decontamination, and disposal of PPE.

Highspeed handpieces are the most important tools in a dental office [32]. When it comes to cleaning handpieces, although contamination decreases with internal irrigation and external disinfection, this is not enough. Sterilization of handpieces is essential for dental practices to provide safe care [33]. In the dental office, it is important to avoid cross-contamination by implementing effective hygiene measures and infection control procedures [34]. High-level disinfection is quick, effective, inexpensive, and recommended whenever heat sterilization is not feasible, which is not the case for handpieces [35]. The dental staff must be aware of the differences between the two procedures, which is essential in infection control in dentistry, and cleaning and decontamination are mandatory steps [36].

The aim of the present study was to assess the knowledge of dental students and young dentists in Romania regarding infection control, including means of infection transmission, personal protective equipment, and methods of disinfection and sterilization of dental instruments.

## 2. Materials and Methods

The questionnaire method was used for the assessment of the students’ level of knowledge.

### 2.1. Setting and Participants

A cross-sectional, questionnaire-based online survey was conducted from March 2021 to February 2022 during the SARS-CoV-2 pandemic, a critical period, to evaluate how prepared medical students were to respond to this public health emergency. Participants were students currently enrolled at the medical dental school in Iași, Romania. According to the secretary of the Faculty of Dental Medicine within the “Grigore T. Popa” University of Medicine and Pharmacy from Iași, the total population of dental medical students in the dentistry program conducing to a Bachelor of Dentistry in Romanian language degree was 936 students [37]. The calculated sample size was made with a formula for confidence level of p = 95%, z = 1.96, with margin of error by 8%, by population size N = 936 [38]. The resulting calculated sample size was 130 students. The selected sample was representative for the university. A total of 150 students were included in the study sample. Participant sampling was volunteer based. The study had a very low level of statistical power alpha, α < 0.5. The survey was sent to eligible participants by the coauthors using the Google Docs platform. The participants were encouraged to roll out the survey among other dental medical students.

### 2.2. The Survey

A 21-item online questionnaire was used to collect data about dental students’ knowledge on infection control. The questionnaire was reviewed for face validity by three experts in dental medical education to identify key issues that may be relevant to dental medical students to assess its relevance and accuracy. During this process, students completed the survey in full and then were interviewed by three members (S-C. I; B-L; D-A) of the research team to elicit their feedback and suggestions for improvement. The 20 students who completed the pilot-testing did not participate in the final survey and the responses collected during pilot-testing were not included in the final analysis. The questionnaire was openly applied and it was uploaded online on the Google Docs platform. The questionnaire included 1 closed-answer question and 20 multiple-answers questions.

### 2.3. Study Group

The study included dental students and resident dentists randomly selected from the “Grigore T. Popa” University of Medicine and Pharmacy in Iași. The selection of the study group was made following selection criteria in accordance with ethical rules and good practices of study. Ethical acceptance for these questions was given in No. 145/31.01.2022. The inclusion criteria were: students enrolled in a form of education with a medical profile, subjects trained for or working in the field of dentistry; resident dentists; subjects who gave their consent to participate in the study; students who agreed to fill in the questionnaire; students attending years I to VI. The students’ answers were organized in groups, with each group including two years of study, for a clearer and more concise presentation of the results: I and II-year students (the preclinical training stage), III- and IV-year students (who are at the beginning of clinical training), V- and VI-year students (who perform clinical activities and are in the final years of study), and resident dentists who are young graduates.

The exclusion criteria were: students who did not agree to participate in the study; students not attending a university with a medical profile. The students considered eligible were those who agreed to complete the questionnaire after reading its contents. A total of 150 subjects completed the questionnaire.

### 2.4. Demographic Characteristics

Demographic data were collected from respondents, including: age, gender, year of study in the university. The 3 items referred to demographic data (Q1–Q3).

### 2.5. Domain: Knowledge Data

The knowledge domain was composed of 18 questions evaluating students’ knowledge about infection control. For each question in this domain, respondents had multiple answer options. The 18 items (Q4–Q21) referred to data on infection control as follows: Q4–Q5 = The modes of infection transmission in the dental office—multiple choice (3–5 options); Q6–Q7 = The importance of hand hygiene and the limitations of using hydroalcoholic solutions for hand hygiene—multiple choice (3 options); Q8–Q12 = Airborne infection transmission—multiple choice (2–7 options); Q13–Q17 = Bloodborne infection transmission—multiple choice (2–5 options); Q18–Q20 = Infection control of hand pieces and sterilization—multiple choice (2–5 options); Q21 = How they gained the information—multiple choice (6 options).

### 2.6. Data Collection

A descriptive statistic of the study was performed by applying crosstabs to all the aspects analyzed according to students and postgraduates. The chi-square test was used for data comparison. Symmetric measurements were performed as follows: Nominal by Nominal (Phi, Cramer’s V, Contingency Coefficient), Interval by Interval (Pearson’s R), and Ordinal by Ordinal (Spearman Correlation). The data were analyzed using IBM-SPSS version 26 (IBM, Armonk, NY, USA), and *p* ≤  0.05 was considered statistically significant.

## 3. Results

### 3.1. Demographic Data

The study sample included 150 subjects: 32% (48) resident dentists and 68% (102) dental students from Iași, Romania, with a mean age of 25.71 ± 4.54 years. The distribution by gender was 75.3% (113) female and 24.7% (37) male.

The distribution of students according to the year of study was as follows: 12% (18) from years I–II, 14.67% (22) from years III–IV, 40.66% (61) from years V–VI, and 32.67% (49) resident dentists (Table 1).

### 3.2. Knowledge Level about the Modes of Infection Transmission in the Dental Office

The modes of infection transmission in the dental office were fully known by 74% (111) of the subjects. A percentage of 9.33% (14) of the subjects considered only direct and indirect contact to be a mode of transmission, 3.33% (5) considered only airborne transmission, and 0.66% (1) considered only bloodborne transmission. No statistically significant differences were found by gender or year of study (*p* > 0.05). Only 76% (114) of the study subjects were aware that the same measures for infection control should be taken, regardless of the general condition of the patient, as recommended by the protocols of Universal Precautions. Significant differences were found by year of study (*p* = 0.012) (Table 2).

### 3.3. Knowledge Level on Hand Hygiene in Infection Control

Most subjects agreed that hand hygiene is very important in infection control: 96.66% (145). However, many of them did not know the details of the effectiveness of this method: 10% (15) of subjects incorrectly answered that the use of hydroalcoholic solutions does not remove transient flora, and 65.33% (98) of subjects believed that it does not remove resident flora. However, 24.66% (37) of subjects correctly stated that hand hygiene with hydroalcoholic solution cannot remove organic matter. Comparative analysis of the answers by gender and year of study did not show statistically significant differences (*p* = 0.819) (Table 3).

### 3.4. Knowledge Level on Airborne Infection Control

The forms of airborne infection transmission in the dental office were fully known by 54.66% (82) of the subjects. The other respondents considered only one form of airborne infection—aerosols, dried nuclei, or evaporated droplets: 40.66% (61), 0.70% (1), and 4% (6), respectively. Comparative analysis by gender and year of study did not show statistically significant differences (*p* > 0.05) (Table 4).

Assessment of knowledge on aerosol transmission during dental treatments showed that 74% (111) of subjects believed that aerosols are released in the form of a visible cloud during various procedures, like ultrasonic scaling, and through the use of water-cooled turbines; air-flow prophylaxis devices; and air/water spray. A percentage of 2% (3) of subjects believed that aerosols are released only during ultrasonic scaling, 2% (3)—only during the use of the water-cooled turbine, 4.67% (7)—only when using the air-flow prophylaxis device, and 2% (3)—only while using the air/water spray. Incorrectly, 2% (3) of subjects answered that aerosols are released during tooth extractions and during periodontal probing. A percentage of 2% (3) of subjects chose all options as correct. Comparative analysis by variables showed statistically significant differences (*p* = 0.001) by year of study, with a positive correlation (*r* = 0.130) (Table 4).

The ideal method of operating field isolation through the use of rubber dams was known to 82% (123) of study participants, and 55.33% (83) of subjects declared they use preprocedural mouthrinses with antiseptic solutions, with no significant differences by gender or year of study.

A percentage of 48% (72) of subjects incorrectly believed that the maximum efficiency of a protective mask lasts for 1 h, and only 42.66% (64) answered correctly (30 min). Significant differences were found by year of study (*p* = 0.001), with a positive correlation (*r* = 0.026) (Table 4).

### 3.5. Knowledge Level and Attitudes about Bloodborne Infection Control

The transmission of bloodborne pathogens in the dental office was known by only 35.33% (53) of subjects, who indicated HBV (hepatitis B virus), HCV (hepatitis C virus) and HIV (human immunodeficiency virus) as bloodborne pathogens. Erroneously, 16.66% (25) included HAV (hepatitis A virus), 23.3% (35) included HAV and influenza virus, and 23.33% (35) included HAV, HBV, HCV, HIV, influenza virus, and TBC (Mycobacterium tuberculosis). No statistically significant differences were found by gender or year of study (*p* > 0.05) (Table 5).

Regarding the means of transmission of the hepatitis B virus, only 18% (27) of subjects answered that it was only by blood, and 1.33% (2) only perinatally and sexually. Various percentages of respondents incorrectly indicated other means of transmission, such as by sneezing (0.66% (1)), air (2% (3)), or combined variants, in total, 78% (117). Comparative analysis by variables showed statistically significant differences (*p* = 0.032) by the year of study, with a positive correlation (*r* = 0.160) (Table 5).

Most of the subjects were not vaccinated against hepatitis B (*p* = 0.002, *r* = −0.274), although 19.33% (29) of them suffered one injury with used and possibly contaminated sharp instruments, and 18.66% (28) suffered at least two such injuries. When asked if they would be willing to provide dental interventions to patients infected with HBV, HCV, HIV/AIDS, 29.33% (44) said that they would accept only in case of emergency, and 4% (6) said that they would accept. Significant differences were found by year of study (*p* = 0.01, *r* = −0.0109) (Table 5).

### 3.6. Knowledge Level on Infection Control of Hand Pieces and Sterilization

Assessment of knowledge regarding disinfection and sterilization showed that 19.5% (13) of subjects confuse high-level disinfection with sterilization. Only 22% (33) of subjects knew the correct methods for handpiece sterilization, and, of these, 12% (18) were resident dentists (*p*= 0.048, *r* = −0.071). A percentage of 22.66% (34) of subjects did not know that autoclave uses wet heat for instrument sterilization (*p* = 0.193), most of them (12.66% (19)) in V-VI year of study (Table 6).

### 3.7. Results on Information Sources

When asked where they received the most information about infection transmission, prevention, and control in the dental office, 31.33% (47) of subjects indicated university courses and seminars, 0.66% (1) only from courses and seminars held at other institutions, 0.66% (1) stated that they received most information only from the websites of professional organizations, such as the World Health Organization, and 1.33 % (2) indicated published scientific articles. A percentage of 1.33 % (2) said they found information on social media, and 16% (24) found information on TV/radio. Most subjects (64.66% (97)) said that they received the most information about infection control in dentistry from all the above-mentioned sources. No significant differences were found by gender or year of study (*p* > 0.05) (Table 7).

## 4. Discussion

Universal Precautions were introduced by the Centers for Disease Control (CDC) in 1985, and Standard Precautions later in 1995, as a standard set of guidelines to prevent the transmission of bloodborne pathogens and other potentially infectious materials. These guidelines also introduced three transmission-based precautions: airborne, drip, and contact [39].

The results of the present study showed that the subjects included knew how infection is transmitted and knew the conditions for applying the Universal Precautions protocol. These results are consistent with the results of other studies in the field. A cross-sectional study conducted in Saudi Arabia on 318 subjects showed an average level of knowledge of 51.6% of the subjects, but a good level of attitude for 92.1% [40]. Another study conducted in Saudi Arabia on 195 subjects showed a moderate level of knowledge (58.2%) and a good level of attitude (80%) [41].

Cross-infection can be defined as the transmission of infectious agents between patients and healthcare professionals. The Standard Precautions are intended to ensure a safe working environment and to prevent the transmission of occupational and nosocomial infections among dental clinic staff and patients. Awareness and adherence to these recommendations are crucial for the prevention of infections. Similar studies have been conducted worldwide to investigate the knowledge and practices of dental students about infection control, and there is a general consensus that they need to be more aware so that they can be protected from the risks of transmitting infections [42].

According to the current literature, dental students are most likely to come in contact with infectious agents due to contact with blood and other fluids. A study conducted in Lima, Peru, assessed the level of knowledge and attitudes among 347 dental students at the Lima Norte and Chorrillos campuses. The results showed a low level of knowledge among the assessed students, and this topic needs to be addressed so that dental students are aware of the importance of the risks of contacting infections both inside and outside the dental office [43].

PPE protects employees from exposure by creating a barrier against bloodborne pathogens. Basic PPE, including gloves, masks, and gowns, should be readily available and worn whenever there is potential for contact with contaminated body fluids and equipment [31].

Along with PPE, proper hand hygiene is one of the most effective means of control and prevention of disease transmission. All health employees who provide care must perform hand hygiene. The current CDC guidelines recommend using an alcohol-based hand scrub with at least 60% alcohol (60% ethanol or 70% isopropyl alcohol) or washing hands with soap and water for at least 20 s before and after touching a patient or performing an aseptic procedure. Hand hygiene should also be practiced when moving from a dirty place to a clean place, after touching a patient, contact with blood, body fluids or contaminated surfaces. Proper hand hygiene is required immediately before the application and immediately after removal of the PPE [31].

Although most of the subjects in the present study agreed on the importance of hand hygiene in infection control, only a quarter of them had knowledge on the details of its effectiveness. Similar results were found by a study in Germany, where 17% of dental students in years 4 and 5 correctly answered questions concerning hand hygiene [44].

One of the most important methods of reducing airborne pathogen transmission in the dental office is to use preprocedural mouthrinses with antiseptic solutions. From this point of view, results similar to the present study were found by a study conducted in Saudi Arabia among dental students in years 3, 4 and 5, who stated, in a percentage of 55%, that they use this method of infection control (55.33% in the present study) [45].

The standard for bloodborne pathogens requires employers to provide workers with the hepatitis B vaccination series, free of charge, within 10 days of the employee’s appointment and after mandatory training on bloodborne pathogens. The vaccination series, usually given in three or four injections over a period of six months, must be given to the worker at a reasonable time and place. Employees have the right to refuse vaccination, but must sign a declination form stating this [31]. The vaccination rate among the subjects of the present study was low (40%) compared to the ones found by studies developed in India and Saudi Arabia on third-, fourth-, and fifth-year dental students (93.40% and 95.4%, respectively) [5,46].

Careful assessment of the exposure and the source of exposure should be made immediately after exposure. The employee’s medical evaluation should be performed immediately, as some treatment decisions, including chemoprophylaxis, should be made within 2 h of exposure. Follow-up assessments should take place at an occupational clinic in one week, three months, six months, and twelve months, depending on the type and source of exposure.

To the question assessing the knowledge of HBV transmission routes, similar studies on the transmission of the infection by blood had a lower rate of correct responses compared to our study, in which 86.7% answered that HBV is transmitted by blood and 79% for HCV. Thus, in a study conducted at Punjab Hospital in Pakistan, 76.2% of the participants in the study answered that HBV is transmitted by blood [47].

In a study conducted at the Vardhman Institute of Medical Sciences, Pawapuri, Bihar, India, 89% of the students surveyed believed that HCV is also transmitted through blood, 97% of the students surveyed thought that HBV is transmitted through infected transfused blood, 96% believed it followed a sting with a used and infected sharp instrument, 87% knew about perinatal transmission, 93.5% knew about transmission by sexual contact, and 84.5% answered that the transmission of the hepatitis B virus can also take place through tattoos or piercings [48].

Regarding where they received the most information about the routes of transmission of the infection, as well as methods for its prevention and control, a study was conducted in March 2020 at the Faculty of Dentistry at Firat University, and an online questionnaire was filled out by 355 dentistry students [49]. The results of their study were as follows: only 25.1% said they took part in seminars on COVID-19 organized by the university to which they belong; 75.8% mentioned that they obtained information about SARS-CoV-2 infection from the websites or socialization accounts of professional organizations, such as the Ministry of Health, the Association of Dentists, and WHO; 21.9% attended seminars held in other institutions; 29.2% read published scientific articles; 41.4% read individual medical sites or social media accounts such as Instagram, Facebook and Twitter; 64.8% obtained information from television and radio programs; and 65.3% from communication groups such as WhatsApp or Line [49].

The clinical relevance of the present study lies in the fact that a lack of knowledge in any assessed aspect materializes in carrying out a clinical activity in conditions of high biological risk, with a lack of control in infection transmission. This study had some limitations that need to be taken into consideration: the small number of participants, the uneven distribution by gender and by year of study or specialization, the random selection of the subjects, and lack of bias assessment.

## 5. Conclusions

Within the limitations of this study, we can conclude that infection transmission in the dental office was fully known by 74% of the subjects.

Although the subjects know the importance of hand hygiene, they do not know that the use of hydroalcoholic solutions removes transient microbial flora and the resident microbial flora.

The control of airborne infection in the dental office was known by only 54.66% of the subjects.

Assessment of knowledge about the transmission of aerosols during dental treatments showed that 74% of the subjects believed that aerosols were released as a visible cloud in combined variants.

Transmission of bloodborne pathogens in the dental office was known by only 35.3% of the subjects. Only one third of the participants knew that HIV, HBV, and HCV are blood-transmitted. Although the incidence rate of occupational accidental injuries is quite high, not all subjects know all the aspects related to bloodborne infection control in the dental office. More than half of the subjects were not vaccinated against hepatitis B.

A percentage of 19.5% of subjects confused the notion of high-level disinfection with sterilization.

Only 22% of subjects knew the methods of sterilization for handpieces, and 12% of them were resident dentists.

Furthermore, 22.66% of the subjects did not know the type of heat used for autoclave sterilization, the majority (12.66%) from study years V–VI.

The sources of information were diverse for 64.66% of respondents, and only from courses and seminars for 31.33% of them.

Taking into account the issues discussed, the promotion of infection control methods, risk awareness courses, and programs, as well as courses to motivate the application of universal precautions, should be a priority. There is need for to improve the level of knowledge for both dental students and young dentists, as infection control is essential in their working field.

## Figures and Tables

**Table 1:** Subject distribution by year of study and gender.

		Year of Study					
		I–II	III–IV	V–VI	R	Total	
		*n* (%)	*n* (%)	*n* (%)	*n* (%)	*n*	%
Gender	F	4 (2.67)	5 (3.33)	14 (9.33)	14 (9.33)	37	24.66
	M	14 (9.33)	17 (11.34)	47 (31.33)	35 (23.34)	113	75.33
Total		18 (12)	22 (14.67)	61 (40.66)	49 (32.67)	150	100.00

*n* = count; F = female; M = male; R = resident dentists.

**Table 2:** Frequency distribution of answers to the questions about the modes of infection transmission in the dental office by gender and year of study.

Answer Options					Year of Study							
	F	M	*p*	*r*	I–II	III–IV	V–VI	R	*p*	*r*	Total	
					*n*	*n*	*n*	*n*			*n*	*%*
Q4 = What are the modes of infection transmission in the dental office?												
Direct and indirect contact	3	11	0.59	−0.05	4	2	5	3	0.70	0.11	14	9.33
Blood transmission	0	1			0	0	1	0			1	0.66
Air transmission	2	3			0	0	2	3			5	3.33
All options	25	86			12	16	47	36			111	74.00
Combined answers	7	12			2	4	6	7			19	12.70
Total	37	113			18	22	61	49			150	100.00
Q5 = Which patients require more attention to be paid for infection control?												
Patients suffering from influenza, measles, enterocolitis	0	1	0.41	−0.11	0	0	1	0	0.01	0.00	1	0.66
Patients infected with HIV, HBV, HBC, TB	6	29			3	2	25	5			35	23.33
All patients	31	83			15	20	35	44			114	76.00
Total					18	22	61	49			150	100.00

*n* = count; R = resident dentists; *p* = Significance level; *r* = Pearson’s correlation coefficient.

**Table 3:** Frequency distribution of answers about the importance of hand hygiene and the limitations of using hydroalcoholic solutions by gender and year of study.

Answer Options					Year of Study							
	F	M	*p*	*r*	I–II	III–IV	V–VI	R			Total	
	*n*	*n*			*n*	*n*	*n*	*n*	*p*	*r*	*n*	*%*
Q6 = How important do you consider hand washing after each patient?												
Unimportant, as long as protective gloves have been used	0	1	0.85	−0.03	0	0	0	1	0.00	0.35	1	0.66
Important, but not mandatory	1	3			1	1	0	2			4	2.66
Very important	36	109			17	21	61	46			145	96.66
Total	37	113			18	22	61	49			150	100.00
Q7 = What are the limitations of using hydroalcoholic solutions for hand hygiene?												
Does not remove transient flora	2	13	0.18	−0.15	2	2	5	6	0.82	−0.01	15	10.00
Does not remove resident flora	22	76			13	12	42	31			98	65.33
Does not remove organic matter	13	24			3	8	14	12			37	24.66
Total	37	113			18	22	61	49			150	100.00

*n* = count; R = resident dentists; *p* = significance level; *r* = Pearson’s correlation coefficient.

**Table 4:** Frequency distribution of answers to the questions about air contaminants and infection control by gender and year of study.

Answer Options					Year of Study							
	F	M	*p*	*r*	I–II	III–IV	V–VI	R	*p*	*r*	Total	
	*n*	*n*			*n*	*n*	*n*	*n*			*n*	*%*
Q8 = What are the forms of air contaminants?												
Aerosols	17	44	0.5	0.05	9	7	24	21	0.83	0.00	61	40.66
Dried nuclei	0	1			0	0	1	0			1	0.66
Evaporated droplets	0	6			0	1	4	1			6	4.00
All the above options	20	62			9	14	32	27			82	54.66
Total	37	113			18	22	61	49			150	100.00
Q9 = Which dental procedures often release aerosols in the form of a visible cloud?												
Ultrasonic scaling	5	15	0.6	0.02	4	1	10	5	0.00	0.13	20	13.33
Use of water-cooled turbine	0	3			3	0	0	0			3	2.00
Tooth extraction, periodontal probing	1	2			1	0	2	0			3	2.00
Use of air-flow prophylaxis device	2	5			0	1	5	1			7	4.67
Use of air/water spray	2	1			2	0	0	1			3	2.00
Ultrasonic scaling; water-cooled turbine; air-flow prophylaxis device; air/water spray	27	84			7	20	43	41			111	74.00
All options	0	3			1	0	1	1			3	2.00
Total	37	113			18	22	61	49			150	100.00
Q10 = Do you consider the rubber dam to have a role in preventing the spread of infections?												
Yes	30	93	0.5	−0.01	13	16	49	45	0.12	−0.19	123	82.00
No	7	20			5	6	12	4			27	18.00
Total	37	113			18	22	61	49			150	100.00
Q11 = Do you use patient preprocedural mouthrinses with antiseptic solutions before starting a dental procedure?												
No, I don’t	4	19	0.7	−0.07	3	2	10	8	0.96	−0.06	23	15.33
Sometimes	11	33			4	7	17	16			44	29.33
Yes, I do	22	61			11	13	34	25			83	55.33
Total	37	113			18	22	61	49			150	100.00
Q12 = How long does the maximum efficiency of the protective mask last?												
1 h	19	53	0.6	0.06	11	4	31	26	0.00	0.03	72	48.00
30 min	16	48			6	16	29	13			64	42.66
As long as it is not damaged	2	12			1	2	1	10			14	9.33
Total	37	113			18	22	61	49			150	100.00

*n* = count; R = resident dentists; *p* = significance level; *r* = Pearson’s correlation coefficient.

**Table 5:** Frequency distribution of answers to the questions about bloodborne infection control by gender and year of study.

Answer Options					Year of Study							
	F	M	*p*	*r*	I–II	III–IV	V–VI	R	*p*	*r*	Total	
	*n*	*n*			*n*	*n*	*n*	*n*			*n*	*%*
Q13 = What pathogens can be blood-transmitted during dental procedures?												
HBV	0	2	0.43	−0.11	1	0	0	1	0.16	0.00	2	1.33
HCV	1	0			1	0	0	0			1	0.66
Influenza virus	0	2			1	0	1	0			2	1.33
TBC	1	1			1	1	0	0			2	1.33
HIV	0	6			1	1	4	0			6	4.00
HBV, HCV, HIV	12	41			1	8	23	21			53	35.33
HBV, HIV	2	9			1	1	6	3			11	7.33
HBV, HCV, HIV, TBC	3	8			1	0	6	4			11	7.33
HAV, HBV, HCV, HIV	5	20			4	5	7	9			25	16.66
HBV, HCV, HIV, influenza virus	1	1			0	0	2	0			2	1.33
HAV, HBV, HCV, HIV, influenza virus, TBC	12	23			6	6	12	11			35	23.33
Total	37	113			18	22	61	49			150	100.00
Q14 = What are the means of transmission of hepatitis B virus?												
Perinatally, sexually	1	1	0.87	0.00	1	1	0	0	0.03	0.17	2	1.33
By sneezing	0	1			1	0	0	0			1	0.66
By blood	6	21			3	4	14	6			27	18.00
By air	1	2			0	2	1	0			3	2.00
Combined variants	29	88			13	15	46	43			117	78.00
Total	37	113			18	22	61	49			150	100.00
Q15 = Are you vaccinated against hepatitis B?												
Yes	15	45	0.54	0.01	2	9	20	29	0.00	−0.27	60	40.00
No	22	68			16	13	41	20			90	60.00
Total	37	113			18	22	61	49			150	100.00
Q16 = Have you ever suffered an injury while performing a dental operation with used and possibly contaminated sharp instruments?												
Yes, but only once	8	21	0.91	0.04	0	1	13	15	0.00	−0.35	29	19.33
Yes, twice or more times	7	21			2	4	7	15			28	18.66
No, I have never suffered such an injury	22	71			16	17	41	19			93	62.00
Total	37	113			18	22	61	49			150	100.00
Q17 = Do you accept to provide dental interventions to patients infected with HBV, HCV, HIV/AIDS?												
Yes, but only in case of emergencies	8	36	0.41	−0.07	2	4	28	10	0.01	−0.11	44	29.33
Yes, I do	28	72			15	16	30	39			100	66.66
No, I do not accept	2	5			1	2	3	0			6	4.00
Total	38	113			18	22	61	49			150	100.00

*n* = count; R = resident dentists; *p* = significance level; *r* = Pearson’s correlation coefficient.

**Table 6:** Frequency distribution of answers to the questions about infection control of hand pieces and disinfection and sterilization by gender and year of study.

Answer Options					Year of Study							
	F	M	*p*	*r*	I–II	III–IV	V–VI	R			Total	
	*n*	*n*			*n*	*n*	*n*	*n*	*p*	*r*	*n*	*%*
Q18 = What is the difference between sterilization and high-level disinfection?												
Sterilization destroys all microorganisms, including spores, which is not done by high-level disinfection	34	103	0.57	0.01	14	21	54	48	0.05	−0.17	137	91.33
High-level disinfection destroys all microorganisms, including spores, which is not done by sterilization	3	10			4	1	7	1			13	8.66
Total	37	113			18	22	61	49			150	100.00
Q19 = How can handpieces be sterilized?												
By wet heat	8	25	0.99	0.01	2	6	7	18	0.05	−0.17	33	22.00
By dry heat	5	12			0	2	10	5			17	11.33
In solvents	2	7			3	1	4	1			9	6.00
In the ultrasound bath	2	6			0	1	5	2			8	5.33
By all the above methods	20	63			13	12	35	23			83	55.33
Total	37	113			18	22	61	49			150	100.00
Q20 = What kind of heat does the autoclave use for instruments sterilization?												
Dry heat	7	27	0.35	−0.05	4	4	19	7	0.19	0.05	34	22.66
Wet heat	30	86			14	18	42	42			116	77.33
Total					18	22	61	49			150	100.00

*n* = count; R = resident dentists; *p* = Significance level; *r* = Pearson’s correlation coefficient.

**Table 7:** Frequency distribution of answers to question Q21 = “Where did you get the most information about infection transmission, prevention and control?” by gender and year of study.

Answer Options					Year of Study							
	F	M	*p*	*r*	I–II	III–IV	V–VI	R	*p*	*r*	Total	
	*n*	*n*			*n*	*n*	*n*	*n*			*n*	*%*
University courses and seminars	9	38	0.69	−0.09	4	13	21	9	0.31	0.11	47	31.33
Courses and seminars held within other institutions	0	1			0	0	1	0			1	0.66
Websites of professional organizations such as WHO	0	1			0	0	0	1			1	0.66
Published scientific papers	1	1			0	0	1	1			2	1.33
Social media accounts	1	1			0	0	1	1			2	1.33
All	26	71			14	9	37	37			97	64.66
Total	37	113			18	22	61	49			150	100.00

*n* = count; R = resident dentists; *p* = Significance level; *r* = Pearson’s correlation coefficient.

## Data Availability

The data that support the findings of this study are available on request from the corresponding author.

## References

[1] Sebastiani F.R., Dym H., Kirpalani T. (2017). Infection Control in the Dental Office. Dent. Clin. North Am..

[2] Saveanu C.I., Porsega A., Anistoroaei D., Iordache C., Bobu L., Saveanu A.E. (2022). Cross-Sectional Study to Evaluate Knowledge on Hand Hygiene in a Pandemic Context with SARS-CoV-2. Medicina.

[3] Meslec M.D. (2021). Assessing the Attitude of Young Doctors Towards the Transmission of the Infection in Medicine. License Thesis.

[4] Porsega A. (2021). Assessment of Student Knowledge, Behavior and Attitudes in Relation to Hand Decontamination. License Thesis.

[5] AL-Essa N.A., Al Mutairi M.A. (2017). To what extent do dental students comply with infection control practices?. Saudi J. Dent. Res..

[6] Ibrahim N.K., Alwafi H.A., Sangoof S.O., Turkistani A.K., Alattas B.M. (2017). Cross-infection and infection control in dentistry: Knowledge, attitude and practice of patients attended dental clinics in King Abdulaziz University Hospital, Jeddah, Saudi Arabia. J. Infect. Public Health.

[7] Algaissi A.A., Alharbi N.K., Hassanain M., Hashem A.M. (2020). Preparedness and response to COVID-19 in Saudi Arabia: Building on MERS experience. J. Infect. Public Health.

[8] Volgenant C., de Soet J.J. (2018). Cross-transmission in the Dental Office: Does This Make You Ill?. Curr. Oral Health Rep..

[9] Rotter M., Mayhall C.G. (1999). Hand washing and hand disinfection. Hospital Epidemiology and Infection Control.

[10] Centers for Disease Control and Prevention: Show Me the Science—When&How to Use Hand Sanitizer in Community Settings. https://www.cdc.gov/handwashing/show-me-the-science-hand-sanitizer.html.

[11] World Health Organization Hand Hygiene—Why, How and When? WHO 2009. https://www.who.int/gpsc/5may/Hand_Hygiene_Why_How_and_When_Brochure.pdf.

[12] Nuwagaba J., Rutayisire M., Balizzakiwa T., Kisengula I., Nagaddya E.J., Dave D.A. (2021). The Era of Coronavirus: Knowledge, Attitude, Practices, and Barriers to Hand Hygiene Among Makerere University Students and Katanga Community Residents. Risk Manag. Healthc. Policy.

[13] UNICEF Everything You Need to Know about Washing Your Hands to Protect Against Coronavirus (COVID-19). Washing Your Hands Can Protect You and Your Loved Ones. https://www.unicef.org/coronavirus/everything-you-need-know-about-washing-your-hands-protect-against-coronavirus-covid-19.

[14] Ejemot-Nwadiaro R.I., Ehiri J.E., Arikpo D., Meremikwu M.M., Critchley J.A. (2015). Hand washing promotion for preventing diarrhoea. Cochrane Database Syst. Rev..

[15] Curtis V., Cairncross S. (2003). Effect of washing hands with soap on diarrhea risk in the community: A systematic review. Lancet Infect. Dis..

[16] Stone S., Teare L., Cookson B. (2001). Guiding hands of our teachers. Hand-hygiene Liaison Group. Lancet.

[17] Rahim M.H.A., Ibrahim M.I., Noor S.S.M., Fadzil N.M. (2021). Predictors of Self-Reported Hand Hygiene Performance among Nurses at Tertiary Care Hospitals in East Coast Malaysia. Int. J. Environ. Res. Public Health.

[18] Dwipayanti N.M.U., Lubis D.S., Harjana N.P.A. (2021). Public Perception and Hand Hygiene Behavior During COVID-19 Pandemic in Indonesia. Front. Public Health.

[19] Alsoufi A., Alsuyihili A., Msherghi A., Elhadi A., Atiyah H., Ashini A., Ashwieb A., Ghula M., Ben Hasan H., Abudabuos S. (2020). Impact of the COVID-19 pandemic on medical education: Medical students’ knowledge, attitudes, and practices regarding electronic learning. PLoS ONE.

[20] Cheng H.C., Peng B.Y., Lin M.L., Chen S.L. (2019). Hand hygiene compliance and accuracy in a university dental teaching hospital. J. Int. Med. Res..

[21] Pittet D. (2000). Improving compliance with hand hygiene in hospitals. Infect. Control Hosp. Epidemiol..

[22] Selwyn S. (1980). Microbiology and ecology of human skin. Practitioner.

[23] Scarano A., Inchingolo F., Lorusso F. (2020). Environmental Disinfection of a Dental Clinic during the COVID-19 Pandemic: A Narrative Insight. BioMed Res. Int..

[24] Tysiąc-Miśta M., Dubiel A., Brzoza K., Burek M., Pałkiewicz K. (2021). Air disinfection procedures in the dental office during the COVID-19 pandemic. Medycyna Pracy.

[25] Karuppasamy K., Obuchowski N. (2021). Comparison of Fit for Sealed and Loose-Fitting Surgical Masks and N95 Filtering Facepiece Respirators. Ann. Work. Expo. Health.

[26] Rengasamy S., Eimer B.C., Szalajda J. (2014). A quantitative assessment of the total inward leakage of NaCl aerosol representing submicron-size bioaerosol through N95 filtering facepiece respirators and surgical masks. J. Occup. Environ. Hyg..

[27] Suen L., Guo Y.P., Ho S., Au-Yeung C.H., Lam S.C. (2020). Comparing mask fit and usability of traditional and nanofibre N95 filtering facepiece respirators before and after nursing procedures. J. Hosp. Infect..

[28] Fouladi Dehaghi B., Ghodrati-Torbati A., Teimori G., Ibrahimi Ghavamabadi L., Jamshidnezhad A. (2020). Face masks vs. COVID-19: A systematic review. Investig. Y Educ. En Enferm..

[29] Rengasamy S., Eimer B.C. (2012). Nanoparticle penetration through filter media and leakage through face seal interface of N95 filtering facepiece respirators. Ann. Occup. Hyg..

[30] Seresirikachorn K., Phoophiboon V., Chobarporn T., Tiankanon K., Aeumjaturapat S., Chusakul S., Snidvongs K. (2021). Decontamination and reuse of surgical masks and N95 filtering facepiece respirators during the COVID-19 pandemic: A systematic review. Infect. Control. Hosp. Epidemiol..

[31] Denault D., Gardner H. OSHA Bloodborne Pathogen Standards. In StatPearls. StatPearls Publishing 2021. https://pubmed.ncbi.nlm.nih.gov/34033323/.

[32] Christensen G.J. (1999). The high-speed handpiece dilemma. J. Am. Dent. Assoc..

[33] Sasaki J.I., Imazato S. (2020). Autoclave sterilization of dental handpieces: A literature review. J. Prosthodont. Res..

[34] Laheij A., de Soet J.J., Volgenant C. (2018). Infectie preventie in de praktijk—Het inrichten van de tandartspraktijk [Implementation of effective infection control: Furnishing a dental practice]. Ned. Tijdschr. Voor Tandheelkd..

[35] Muscarella L.F. (1996). High-level disinfection or "sterilization" of endoscopes?. Infect. Control. Hosp. Epidemiol..

[36] Rutala W.A., Weber D.J. (2013). Disinfection and sterilization: An overview. Am. J. Infect. Control..

[37] https://www.umfiasi.ro/ro/academic/programe-de-studii/licenta/Pagini/Student---Medicin%C4%83-dentar%C4%83.aspx.

[38] Xia F., Hughes J.P., Voldal E.C., Heagerty P.J. (2021). Power and sample size calculation for stepped-wedge designs with discrete outcomes. Trials.

[39] Broussard I.M., Kahwaji C.I. (2021). Universal Precautions. StatPearls.

[40] Srivastava K.C., Shrivastava D., Sghaireen M.G., Alsharari A.F., Alduraywish A.A., Al-Johani K., Alam M.K., Khader Y., Alzarea B.K. (2020). Knowledge, attitudes and practices regarding COVID-19 among dental health care professionals: A cross-sectional study in Saudi Arabia. J. Int. Med. Res..

[41] Shrivastava D., Alduraywish A.A., Srivastava K.C., Alsharari A.F., Al-Johani K., Sghaireen M.G., Alam M.K. (2021). Assessment of knowledge and attitude of allied healthcare professionals about COVID-19 across Saudi Arabia. Work.

[42] Qamar M.K., Shaikh B.T., Afzal A. (2020). What Do the Dental Students Know about Infection Control? A Cross-Sectional Study in a Teaching Hospital, Rawalpindi, Pakistan. BioMed Res. Int..

[43] Silva O., Palomino S., Robles A., Ríos J., Mayta-Tovalino F. (2018). Knowledge, Attitudes, and Practices on Infection Control Measures in Stomatology Students in Lima, Peru. J. Environ. Public Health.

[44] Baier C., Albrecht U.-V., Ebadi E., Vonberg R.-P., Schilke R. (2020). Knowledge about hand hygiene in the Generation Z: A questionnaire-based survey among dental students, trainee nurses and medical technical assistants in training. Am. J. Infect. Control..

[45] Alharbi G., Shono N., Alballaa L., Aloufi A. (2019). Knowledge, attitude and compliance of infection control guidelines among dental faculty members and students in KSU. BMC Oral Health.

[46] Deogade S.C., Suresan V., Galav A., Rathod J., Mantri S.S., Patil S.M. (2018). Awareness, knowledge, and attitude of dental students toward infection control in prosthodontic clinic of a dental school in India. Niger. J. Clin. Pract..

[47] Khurram M., Qaisar A., Zafar K.J., Wahid A., Hassan F., Javed M. (2020). Assessment of Knowledge and Attitude towards Hepatitis B Infection Among Dental Students in two Teaching Hospitals of Punjab, Pakistan. Annals Punjab. Med. Coll..

[48] Kumar A., Kumar S., Har T., Kumar L., Kumar V. (2021). KAP of Medical Students towards Hepatitis B and Hepatitis C- A questionnaire-based study. Int. J. Health Clin. Res..

[49] Ataş O., Talo Yildirim T. (2020). Evaluation of knowledge, attitudes, and clinical education of dental students about COVID-19 pandemic. PeerJ.

